# Depression, anxiety, and burnout in academia: topic modeling of PubMed abstracts

**DOI:** 10.3389/frma.2023.1271385

**Published:** 2023-11-27

**Authors:** Olga Lezhnina

**Affiliations:** Learning and Skill Analytics Research Group, TIB Leibniz Information Centre for Science and Technology, Hannover, Germany

**Keywords:** BERTopic, topic modeling, mental health in academia, depression, anxiety, burnout

## Abstract

The problem of mental health in academia is increasingly discussed in literature, and to extract meaningful insights from the growing amount of scientific publications, text mining approaches are used. In this study, BERTopic, an advanced method of topic modeling, was applied to abstracts of 2,846 PubMed articles on depression, anxiety, and burnout in academia published in years 1975–2023. BERTopic is a modular technique comprising a text embedding method, a dimensionality reduction procedure, a clustering algorithm, and a weighing scheme for topic representation. A model was selected based on the proportion of outliers, the topic interpretability considerations, topic coherence and topic diversity metrics, and the inevitable subjectivity of the criteria was discussed. The selected model with 27 topics was explored and visualized. The topics evolved differently with time: research papers on students' pandemic-related anxiety and medical residents' burnout peaked in recent years, while publications on psychometric research or internet-related problems are yet to be presented more amply. The study demonstrates the use of BERTopic for analyzing literature on mental health in academia and sheds light on areas in the field to be addressed by further research.

## 1 Introduction

Mental health disorders are common in the general population, and in academia their prevalence might be even higher (Ibrahim et al., 2013; Müller, 2020). Raising awareness of this problem is an important task that should be addressed by mutual effort of researchers and policy makers (Cahill, 2023). In recent decades, depression, anxiety, and burnout in academia have been increasingly discussed in literature. Human experts can extract valuable insights from analysis of previous findings (Koutsimani et al., 2019; Balhatchet et al., 2023; Ryan et al., 2023), but the growing amount of publications requires more advanced computational methods to process new volumes of the data. Natural language processing (NLP) combines the power of computational linguistics, data science, and computer science to employ machine learning methods for analysis of natural human speech (Albalawi et al., 2020). Topic modeling is a machine learning method in the frame of NLP that aims at finding groups of words (topics) in a corpus of text. It can be categorized as a method of text mining, which is an approach to automatically discover, retrieve, and extract information in a corpus of text involving linguistics, statistics, and computer science (Abbe et al., 2016). In different areas of research and practice, topic modeling is used for uncovering hidden structure of the data, detecting novel and emergent trends, and understanding dynamics of the textual information (Hannigan et al., 2019). For long time, Latent Dirichlet Allocation (LDA, Blei et al., 2003) was the most frequently used method of topic modeling in many areas including mental health research (Moßburger et al., 2020; Park et al., 2021). However, LDA disregards semantic relationships between words, while text embedding techniques allow obtaining a more informative latent semantic structure (Devlin et al., 2019). Recently, BERTopic approach to topic modeling was introduced (Grootendorst, 2022) as a modular technique combining document embedding, dimensionality reduction, density-based clustering, and a weighing scheme that estimates the importance of words in clusters to generate topic-word distributions. This method provides entirely new perspectives on interpreting the topical structure of textual data and outperforms other topic modeling methods (Kukushkin et al., 2022; Udupa et al., 2022).

Topic modeling should be approached from the perspective of its inherent subjectivity: textual data is difficult to analyse due to their varied linguistic characteristics (Blair et al., 2020), and the analysis is “preconfigured through beliefs and values” (Egger and Yu, 2022, p. 2) of researchers. There is a certain degree of discordance between human-actionability and machine-actionability, which is addressed by recent works on cognitive interoperability in knowledge engineering (Vogt et al., 2023) and should be taken into account in topic modeling practice. Previous research (Hannigan et al., 2019; Egger and Yu, 2022) emphasized the role of human judgement influencing analytical decisions in the process of topic modeling.

In this paper, BERTopic is applied to analyzing abstracts of scientific articles on depression, anxiety, and burnout in academia from PubMed published in years 1975–2023. The author does not aim at comparing BERTopic with other approaches but refers the reader to previous studies discussing these comparisons (e.g., de Groot et al., 2022; Egger and Yu, 2022; Kukushkin et al., 2022; Udupa et al., 2022). The main goal of this study is to illustrate the advantages of BERTopic as a modular technique, which allows for flexible choices at each stage of document embedding, clustering, and topic generation, and to provide the reader with a comprehensive description of the process. Analytical decisions made in the study are reported in detail, with the focus on the inevitable influence of the researcher's judgement on these choices. The Python code is in open access on GitHub https://github.com/OlgaLezhnina/BERTopic_academia, so that interested researchers can reproduce the modeling process and the principal findings of the study. Mental health research is still in need of the more extensive use of topic modeling approaches (Zhang et al., 2022), and this paper is intended for specialists widening their repertoire of text mining methods.

## 2 Background

This section contains the outline of recent research on depression, anxiety, and burnout in academia and a brief summary of text mining methods in mental health research. BERTopic as a topic modeling approach is described, and previous studies on its effectiveness are cited.

### 2.1 Current state of research on depression, anxiety, and burnout in academia

Depression is a very common mental disorder, which, according to the World Health Organization (2023), is currently experienced by approximately 280 million people in the world. Diagnostic criteria for depression are listed in the Diagnostic and Statistical Manual of Mental Disorders, Fifth Edition (DSM-5) published by the American Psychiatric Association (APA, 2013) and include, among others, depressed mood, loss of interest and pleasure in all activities, fatigue, and diminished ability to concentrate. Depressed patients are prone to suicidal ideation and have elevated mortality risks (Cuijpers et al., 2014). This disorder has been thoroughly explored by researchers in medicine, psychology, social sciences, and other areas, often in combination with related conditions, such as anxiety and burnout. Anxiety, according to the APA (2023), is a common psychological condition, a normal reaction to stress, which alerts us to danger. Anxiety disorders, in contrast to that, involve excessive or disproportional anxiety and hinder an individual's ability to function. According to the DSM-5, they include generalized anxiety disorder, social anxiety disorder, panic disorder, separation anxiety disorder, substance or medication-induced anxiety disorder, and various phobias (APA, 2013). “State anxiety” as a temporary condition is differentiated from “trait anxiety” as an individual's proneness to anxiety, which is a relatively stable characteristic (see Koutsimani et al., 2019). Researchers and clinicians are in agreement regarding high comorbidity of depression and anxiety (Hirschfeld, 2001), although possible causal relationships between them are still unclear. For instance, Winer et al. (2017) suggested that anxiety and depression are related via anhedonia emerging from the former and resulting in the latter. In the context of professional environments, these two conditions are often accompanied by burnout. Burnout is defined as a disorder composed of emotional exhaustion, depersonalization, and reduced personal accomplishment as a response to chronic emotional and interpersonal stressors in the working environment (Maslach et al., 2001). The degree to which this condition can be differentiated from depression and anxiety is still debated in literature. Koutsimani et al. (2019) in their meta-analysis of 67 published studies explored the relationship between burnout and depression, and the relationship between burnout and anxiety; they came to the conclusion that these are interrelated but distinct conditions.

For long time, research on depression, anxiety, and burnout, as well as on researcher mental health in general, was underrepresented in literature (Guthrie et al., 2017). However, some groups in academia might be at increased risk of these disorders compared to the general population; for instance, a systematic review (Ibrahim et al., 2013) showed a higher prevalence of depression in university students (see Mirza et al., 2021, for the review on medical students). Therefore, the theme of depression, anxiety, and burnout in academia becomes increasingly prominent in mental health research. When Dyrbye et al. (2006) reviewed published papers on depression and anxiety in the US and Canadian medical students, they did not identify studies on burnout in this population. More recent systematic reviews, though, included studies on depression, anxiety, and burnout. Balhatchet et al. (2023) explored burnout and mental wellbeing in Australian postgraduate medical trainees. Ryan et al. (2023) showed that burnout in medical professionals was associated with depression, anxiety, suicidality, and substance abuse. The pandemic times brought changes to academia, such as the increase in burnout (Andrade et al., 2023), which were explored in mental health research. A wider acceptance of internet-based therapy was discussed in literature: Svärdman et al. (2022) conducted a meta-analysis showing effectiveness of internet-delivered cognitive behavioral interventions. The increase in internet addiction was also detected in recent years, and its links to anxiety and depression were explored (Servidio et al., 2021; Gavurova et al., 2022).

In mental health research, advanced methods of data analysis have become widely accepted. Abbe et al. (2016) identified four major areas of text mining application in psychiatry: observational studies focusing on mental disorders; the patients' perspectives and narratives; medical records; and new scientific information in the literature. Shatte et al. (2019) in their systematic review of 300 studies on machine learning methods in mental health research discerned the following areas of application: detection and diagnosis; prognosis, treatment and support; public health; research and clinical administration. Their review included studies on topic modeling for detection of depression. Buddhitha and Inkpen (2023) summarized previous studies on early detection of mental disorders and suicidal ideation. With multi-task learning, they explored the relationship between mental disorders and suicide ideation in Twitter and Reddit datasets. Zhang et al. (2022) in their analysis of 399 published papers on NLP methods for mental disorder detection concluded that most studies relied on supervised learning methods. They urged for a wider use of semi-supervised and unsupervised methods, including topic modeling, and emphasized that interpretability of models is an important factor in mental health research.

### 2.2 BERTopic as a topic modeling approach

Topic modeling is a machine learning and NLP technique aimed at identifying latent topical patterns within collections of texts. Algorithms applied for this purpose include Latent Semantic Analysis, Non-Negative Matrix Factorization (NMF), information-theoretical topic modeling CorEx (Gallagher et al., 2017), and Top2Vec. These approaches use different assumptions that should be taken into account in their application (Schmidt, 2013; Egger and Yu, 2022). The most frequently used method of topic modeling is LDA, a Bayesian technique that employs a generative probabilistic model and Dirichlet distributions to identify topics in the data (Blei et al., 2003). It is the most common topic modeling method in mental health research. For instance, Moßburger et al. (2020) applied LDA to analyse posts in online forums on depression, and Park et al. (2021) to examine the contents of the queries regarding mental disorders in online inquiry platforms. A disadvantage of LDA is that it uses bags-of-words representations and therefore does not capture the context of words (see the summary of LDA critique in Reisenbichler and Reutterer, 2019). This problem is addressed by embedding techniques. Word embedding is a distributed word representation, in which each dimension represents a latent feature of the word aimed at capturing its syntactic and semantic properties (Turian et al., 2010). Recently, Bidirectional Encoder Representations from Transformers (BERT) was introduced as a method of generating contextual word and sentence vector representations (Devlin et al., 2019).

BERTopic (Grootendorst, 2022) is an advanced approach to topic modeling comprising modules that can be flexibly changed depending on the study goals and the dataset characteristics. BERTopic is flexible, does not require preprocessing of the data, and produces interpretable topics generating novel insights (Egger and Yu, 2022). It supports dynamic topic modeling (for dynamic modeling in other approaches, see Alghamdi and Alfalqi, 2015) and class-based topic modeling and allows for multilingual analysis. This method was applied in medicine (Oveh et al., 2022), quality management (Sánchez-Franco et al., 2023), environmental, social, and governance research (Lee et al., 2023), and other areas. In mental health research, BERTopic was applied, for instance, by Cowan et al. (2022) to analyse unstructured speech samples from individuals with serious mental illness, primarily schizophrenia. BERTopic compares favorably with LDA (de Groot et al., 2022; Kukushkin et al., 2022); with Top2Vec and NMF (Egger and Yu, 2022); and with Gibbs Sampling Dirichlet Multinomial Mixture Model (Udupa et al., 2022).

BERTopic modular approach includes a few steps that can be adjusted to the goal of analysis (Grootendorst, 2022). At the first step, the documents are embedded with a pre-trained language model in the frame of Sentence-BERT (SBERT). A specific model can be selected by the user; there are pre-trained multi-lingual language models supporting various languages (e.g., for XLM-RoBERTa model with Thai language, see Munthuli et al., 2023).

The next steps consist of dimensionality reduction and clustering of the embeddings. For dimensionality reduction, Uniform Manifold Approximation and Projection for Dimension Reduction (UMAP) is suggested in the BERTopic approach, although other techniques can be integrated into this stage as well. UMAP is an algorithm based on manifold learning techniques and ideas from topological data analysis (McInnes et al., 2018). Previous studies (e.g., Yang et al., 2021; Kukushkin et al., 2022) confirmed that UMAP is more useful for clustering quality than other methods of dimensionality reduction, such as t-distributed stochastic neighbor embedding (t-SNE). UMAP is a stochastic algorithm as well, which means that for reproducibility of results, a random seed should be fixed. UMAP is effective for finding a low dimensional embedding that preserves the essential topological structure of the data. For a similarity metric, the cosine similarity is typically used. The cosine similarity between two vectors is calculated as follows, where ||·||_2_ denotes the Euclidean norm of a vector:


(1) $$\begin{array}{l} S_{\cos}(\vec{u\;},\vec{v})=\;\frac{\sum_{i=1}^{\left|n\right|}u_{i}*v_{i}}{||\vec{u}|{|}_{2}*||\vec{v}|{|}_{2}} \end{array}$$

In BERTopic, various clustering algorithms can be employed; the suggested option is the Hierarchical Density-Based Spatial Clustering of Applications with Noise algorithm (HDBSCAN). HDBSCAN extends a density-based clustering algorithm DBSCAN with hierarchical clustering, is able to deal with noisy data, and does not assume predefined shape or size of the clusters (Campello et al., 2013; McInnes et al., 2017). Each cluster is assigned one topic. HDBSCAN assigns a document to the outlier category when it does not belong to any of clusters identified by the model.

For word representations of topics, the class-based term frequency inverse document frequency (c-TF-IDF) weighting scheme is applied in BERTopic. This scheme is a modified TD-IDF scheme: the latter assesses representativeness of terms for an individual document, and the former for a topic, which is a cluster of documents. Grootendorst (2022) defines the importance of word *W*_*t, c*_ as the frequency of the term *t* in class *c* multiplied by the inverse class frequency, that is, the logarithm of the average number of words per class divided by the frequency of the term *t* in all classes (here, 1 is added to output only positive values):


(2) $$\begin{array}{l} W_{t,c}=\;tf_{t,c}*\log\left(1+\frac{A}{tf_{t}}\right) \end{array}$$

A model can be fine-tuned with the Maximal Marginal Relevance (MMR) algorithm. MMR diversifies words within documents and thus influences topic interpretability. The diversity parameter of MMR (hereinafter, the keyword diversity) refers to the document and needs to be distinguished from the topic diversity (TD) metric, which is described below.

Topic models can be assessed and compared. Such metrics as topic coherence (TC) and topic diversity (TD) are widely applied for model evaluation (e.g., Blair et al., 2020) but should be understood as “proxies of what is essentially a subjective evaluation” (Grootendorst, 2022, p. 5). TD is calculated as the percentage of unique words in the top n words of all topics. TD value ranges from 0 to 1, with lower values indicating redundant topics, and higher values indicating better diversity (Grootendorst, 2022). The TC metric used in this study is C_v_, which was shown to be the best performing metric in the comprehensive study comparing 237,912 measures on six benchmarks for topic coherence (Röder et al., 2015). To measure coherence of the word set W, word probabilities should be calculated; a confirmation measure should be selected that scores the agreement between two words ω_i_ and ω_j_; and finally, these scalar values for word pairs should be aggregated into a single coherence score. The TC measure C_v_ used in this work compares a word ω_i_ to the total set W. The Boolean sliding window (for C_v_, size 110) moves one word token per step over the documents to create virtual documents to compute word probabilities. For an indirect confirmation measure, C_v_ combines the cosine similarity measure with the normalized pointwise mutual information (NPMI).


(3) $$\begin{array}{l} \text{NMPI}\left(\omega_{\text{i}},\omega_{\text{j}}\right)=\left(\frac{\log\frac{\text{P}\left(\omega_{\text{i}}{\text{, ω}}_{\text{j}}\right)+\varepsilon }{\text{P}\left(\omega_{\text{i}}\right)*\text{P}\left(\omega_{\text{j}}\right)}}{-\log\left(\text{P}\left(\omega_{\text{i}},{\text{ ω}}_{\text{j}}\right)+\varepsilon \right)}\right) \end{array}$$

Thus, the mutual information of two words is calculated; ε is added to avoid the logarithm of zero. To each word ω_i_ we assign a vector with NMPI values as scalar components. The cosine similarities (see Equation 1) are calculated between each word vector and the topic vector, which is the average of the word vectors. The arithmetic mean of the similarity values is taken as the final TC (C_v_) score. The score ranges from 0 to 1, with higher values indicating more coherent topics (Röder et al., 2015).

When the model is selected, the topics can be explored and visualized. In BERTopic, as in any other topic modeling approach, topics can be studied in various ways. To structure these, we can discern three main tasks in exploring the topics. The first is to describe the topics themselves: to label them, to obtain the most representative documents, to list the representative words and visualize them. The second is to explore the topics in relationships with each other: to find similarities between the topics, to visualize them, and to build meta-topic clusters. The third is to trace the topic development with time in the frame of dynamic topic modeling.

For the first task, custom user labels for topics can be assigned based on relevant words and documents of the topic. BERTopic labels the topics with three most frequent terms separated by underscores. However, a researcher might want to use a more intuitive label for a topic. In this case, a larger number of relevant words and relevant documents can be explored, and candidate user-defined labels can be assessed with a BERTopic function that applies bart-large-mnli model for the zero-shot classification task (Grootendorst, 2023). The most frequent terms representing a topic can be obtained and visualized with a bar plot, which shows a selected number of relevant words and their c-TF-IDF scores. The c-TF-IDF score of a word shows its importance for the topic as the cluster of documents (see Equation 2).

The BERTopic visualizations are useful for the second task, exploring similarities between the topics. After the documents were assigned to the topics, we can expect the topics to be sufficiently distinct, and the researcher might decide to merge topics that are too similar. Two BERTopic visualizations are frequently used to show relationships between the topics. The first is the heatmap. It is based on a similarity matrix produced by applying cosine similarities (see Equation 1) through topic embeddings. The second is the hierarchical clustering dengrogram. It presents agglomeration levels of the topics, which are constructed by hierarchical clustering. The clustering is based on the Ward linkage function applied to the cosine distance matrix between topic embeddings. The Ward approach consists in minimizing the merging cost of combining clusters *A* and *B* defined by this formula, in which *x*_*i*_ is a point of a cluster with the center *m*:


(4) $$\begin{array}{l} \Delta \left(A,B\right)=\underset{i\in A\cup B}{\sum }||{\vec{x}}_{i}-{\vec{m}}_{A\cup B}|{|}^{2}-\underset{i\in A}{\sum }||{\vec{x}}_{i}-{\vec{m}}_{A}|{|}^{2}\\ \;-\underset{i\in B}{\sum }||{\vec{x}}_{i}-{\vec{m}}_{B}|{|}^{2} \end{array}$$

Due to the different mathematical underpinnings of the heatmap and the dendrogram, resulting similarities between the topics might differ.

Evolution of topics with time can be explored and visualized in BERTopic. Dynamic topic modeling assumes that topics exist on the whole timespan but their representations change. Therefore, BERTopic first generates the global topic representation by fitting the model on the entirety of documents. Then, the global IDF values are multiplied by term frequency of documents at a given time (Grootendorst, 2022). It means that the word importance score, as defined by Equation 2, is now calculated for each time step *i*:


(5) $$\begin{array}{l} W_{t,c,i}=\;tf_{t,c,i}*\log\left(1+\frac{A}{tf_{t}}\right) \end{array}$$

In this study, BERTopic was selected as the topic modeling method based on previous research showing its effectiveness and benefits of its modular structure. The details of the analysis and the process of model selection are described in the next section.

## 3 Materials and methods

In this section, analytical choices made in the study are explained. The data used for the modeling are briefly described. Finally, the model selection process is outlined.

### 3.1 Analytical strategy

**Figure 1:**
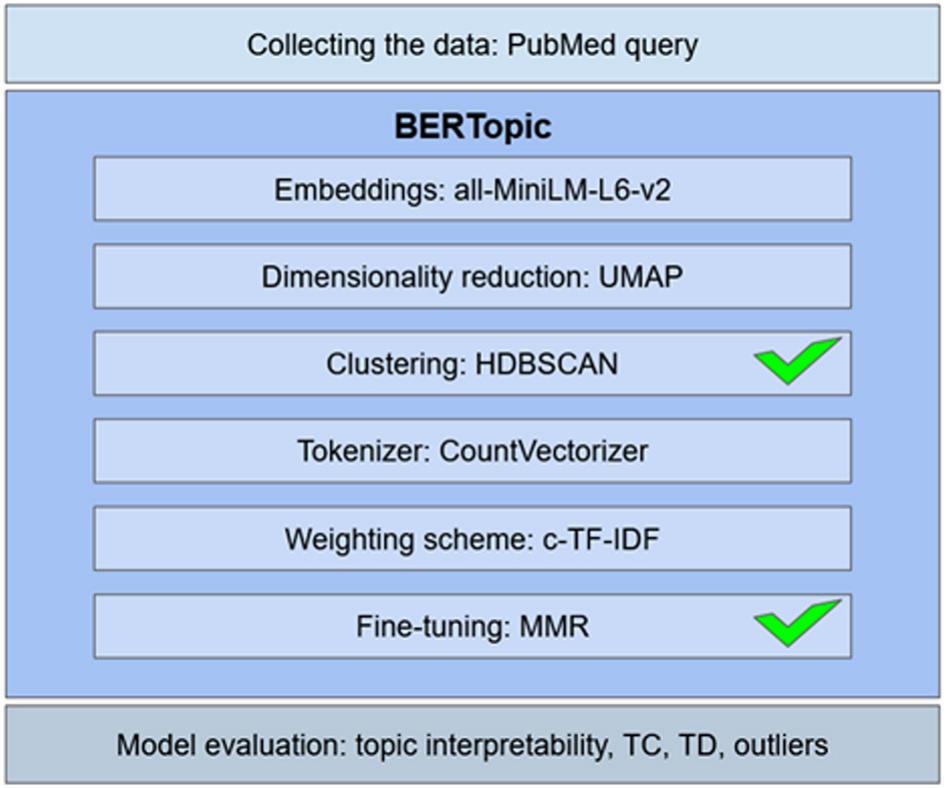
The stages for analytical choices. MMR is Maximal Marginal Relevance, c-TF-IDF is Class-Based Term Frequency Inverse Document Frequency. HDBSCAN is Hierarchical Density-Based Spatial Clustering of Applications with Noise, UMAP is Uniform Manifold Approximation and Projection for Dimension Reduction. The BERTopic modules with parameters that were tuned in this study are marked with ticks.

The results of topic modeling depend on a number of decisions related to selecting model parameters, evaluating the model, and interpreting the results. Figure 1 presents the junctures for analytical choices of the study: the data collection, the BERTopic modules, and the model evaluation.

For information retrieval, the PubMed database as a reliable health-related resource was selected. In this study, abstracts of scientific articles written in English were used. Extracting abstracts rather than full texts is a common practice in topic modeling, as abstracts are informative, concise, and comparable with each other (Daenekindt and Huisman, 2020). The query with the following terms was specified: “depression” or “depressed”, or “depressive”, or “anxiety”, or “burnout”, and “student”, or “research assistant”, or “predoc”, or “postdoc”, or “postdoctoral”, or “PhD candidate”, or “graduate”, or “postgraduate”, or “early career researcher”, or “early-career researcher”, or “early career scientist”, or “early stage researcher” in the title or abstract. At the stage of data collection, different decisions on the database(s) and the query are possible (see the Discussion section).

To analyse the retrieved data, BERTopic modular approach was used, with decisions on each module made in accordance with previous studies and the dataset characteristics. The use of default parameter settings was described as one of the problems in topic modeling (Egger and Yu, 2022); in this regard, BERTopic modules allow for flexible choice of algorithms suitable for specific tasks and for parameter tuning. However, the procedure of model selection and evaluation in topic modeling, including BERTopic, differs from other machine learning techniques and does not imply the grid search of parameters (Grootendorst, 2023).

BERTopic does not require data preprocessing, although it is possible to include such steps as, for instance, removing abbreviations. This additional step was not undertaken because abbreviations in the texts, such as obsessive-compulsive disorder (OCD) or Beck Depression Inventory (BDI), are known to domain specialists; some of them are explained in the Results section. For embeddings, the author resorted to all-MiniLM-L6-v2 model, the recommended BERTopic option. This sentence-transformers model maps sentences and paragraphs to a 384-dimensional dense vector space and is capable of capturing semantic similarities between documents (Grootendorst, 2023). Dimensionality reduction was conducted with UMAP with the default settings 15 nearest neighbors, five components, and the cosine similarity as a metric. A random seed was fixed for the final model. Clustering was conducted with HDBSCAN, with the Euclidian distance as a metric. For HDBSCAN, the minimal cluster size and the minimal number of samples were varying in different models because these two parameters influence topic interpretability and the number of outliers in the model. Topics were tokenized with the default vectorizer model used in BERTopic, as it works effectively with the English language; stop words were removed at this stage. The models were fine-tuned with the MMR using the keyword diversity parameter. Therefore, only two BERtopic modules were used to tune the model parameters: first, the clustering module with the choice of a cluster algorithm and, for HDBSCAN, selection of the minimal cluster size and the minimal number of samples; and second, the keyword diversity in MMR for fine-tuning the model.

Prior to evaluating the BERTopic models, the proportion of outliers was assessed to select a clustering algorithm: if it were high, the solution explored by de Groot et al. (2022) would be possible (with 74% of outliers, they resorted to k-means as a clustering method). In further steps, the proportion of outliers was less important but still had to be considered and, if possible, reduced. For model evaluation, topic interpretability was the main criterion, supplemented with TC and TD metrics.

The topics of the final model are explored and labeled; their relationships with each other visualized; and their evolution traced. In that, the author follows the common BERTopic practices (showing bar plots with c-TF-IDF scores, the heatmap, the hierarchical clustering dendrogram, and the dynamic topic modeling graph) with a few changes. Firstly, the document embeddings are not presented here, as the plot of insufficient resolution would not give full credit to the interactive visualization created by the BERTopic function (see the Python code). Secondly, a simple visualization is added related to associations between a search term and the topics. BERTopic allows finding topics relevant to a search term and gives the similarity score between the term and the topic. The author used NetworkX library in Python to visualize the results of this search as a graph with three relevant topics as nodes. Similarity scores on the edges are cosine similarities between the search term embedding and the topics embeddings. In the study, examples for two search terms are given, and the interested researcher can use the Python code to depict these relationships for any search term.

### 3.2 The data

**Figure 2:**
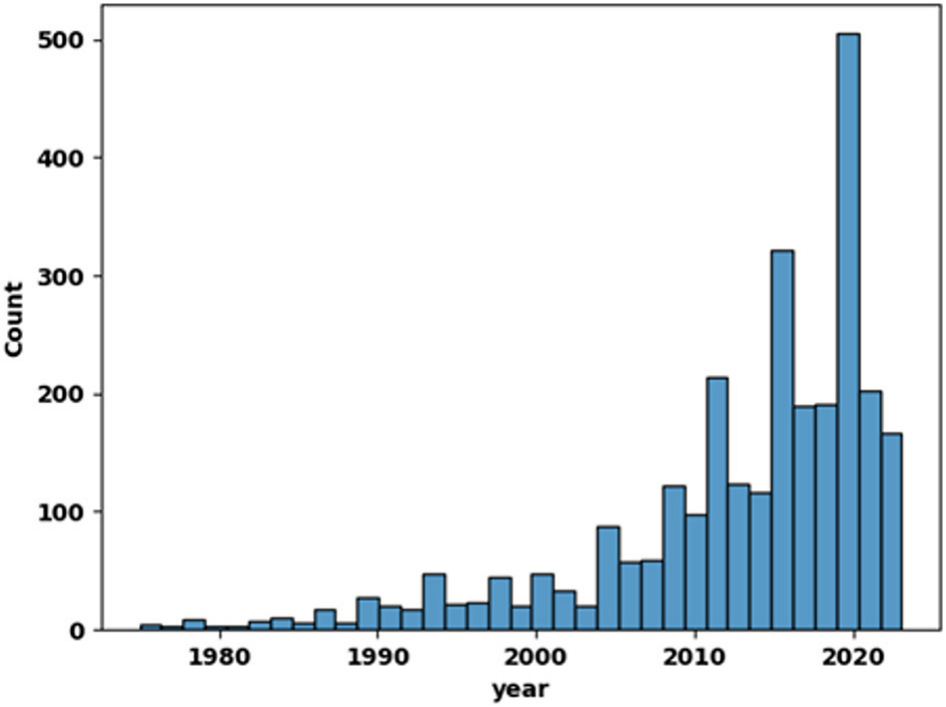
The number of PubMed publications on depression, anxiety, and burnout in academia per year (1975–2023).

Web scrapping from PubMed database was conducted with Python (version 3.9.7, Metapub library). It resulted in the sample of 2,846 abstracts of papers published in 903 journals in years 1975–2023. Prior to the analysis, the data was checked for quality (that is, no missing entries and unique DOIs). The histogram of papers published per year is presented in Figure 2.

### 3.3 Selecting the model

BERTopic models with different sets of parameters were compared in terms of topic interpretability, the number of outliers, TC, and TD. Each of the models was run three times (as in Grootendorst, 2022), with the results presented as a range for the number of topics and the number of outliers, or averaged for TC and TD.

First, the model with the default set of parameters (Model 1) was tested. The default settings in HDBSCAN were minimal cluster size of 15 and minimal sample size of 15; for MMR, the default keyword diversity value was 0.8. Model 1 treated maximum 28% of abstracts as noise. With this proportion of outliers, the use of a different clustering model (such as *k*-means in de Groot et al., 2022) was not required. The model found more than 30 topics in the data, some of which could be seen as redundant. For the next step, Model 2, larger clusters with minimal size of 30 were used to obtain interpretable topics, and minimal number of samples was decreased to 10 in order to further reduce the proportion of outliers. The new model had interpretable clusters, with the proportion of outliers reduced, and performed better in terms of TC. For Model 3, the terms in the topic were diversified: the keyword diversity in MMR was changed from 0.8 to 0.2. TC of Model 3 increased and TD decreased, with the new values that might be perceived as a better balance, and topics were even more interpretable in comparison to Model 2. Finally, an additional increase in the minimal cluster size to 45 was attempted for Model 4. However, this change was not beneficial, as some topics were not identifiable anymore: for instance, a potentially interesting topic on internet-related problems, which persisted in each run of Model 3, was not detectable with this larger cluster size. These considerations led to the choice of Model 3 as the final model. The results are summarized in Table 1.

**Table 1:** BERTopic models.

**Model**	**Parameters**	**N topics**	**Outliers**	**TC**	**TD**	**Interpretability**
Model 1	15, 15, 0.80	32–34	706–757	0.384	0.799	Topics redundant
Model 2	30, 10, 0.80	25–28	575–623	0.404	0.732	Interpretable topics
Model 3	30, 10, 0.20	26–27	659–683	0.536	0.717	Interpretable topics
Model 4	45, 10, 0.20	20–22	632–749	0.543	0.695	Topics underrepresented

The parameters include the minimal cluster size, the minimal number of samples, and the keyword diversity. TC is topic coherence, TD is topic diversity. The results of three runs for each model are given as a range for the number of topics and the number of outliers. For TC and TD, the results are averaged.

The final model was run with the selected parameters of the minimal cluster size 30, the minimal number of samples 10, and the keyword diversity 0.2; the model was assigned a fixed state in UMAP for reproducibility. The model had TC = 0.538 and TD = 0.700. It excluded 600 abstracts (21% of the sample) as mixed or uninterpretable outliers, and the rest were assigned to one of the 27 topics.

## 4 Results

In this section, the final model is described. The identified 27 topics were further explored. Two largest topics had 238 and 223 articles, and two smallest topics had 31 articles each; these smallest topics were not stable in previous runs of Model 3. The topics were labeled based on their representative terms and documents. For instance, the automatically produced label for topic 5 is “depression_bdi_depressive” (BDI is Beck Depression Inventory). The first ten terms for topic 5, with their c-TF-IDF scores, are “depression” (0.035), “bdi” (0.026), “depressive” (0.019), “inventory” (0.018), “psychometric” (0.014), “scales” (0.014), “scores” (0.013), “affective” (0.013), “depressed” (0.012), and “temperament” (0.012). It can be concluded from these terms that the topic is related to psychometric studies exploring measures of depression. The classification score of the user-defined label “psychometrics of depression” (0.995) was high; in comparison, such a label as “depression and anxiety” scored 0.296. Hereinafter, the automatically produced labels are kept for BERTopic visualizations, and a few topics mentioned in the text of the paper are described by their user-defined labels, which were tested with the classifier and obtained scores above 0.990.

For a specific topic, the most representative documents can be found. For instance, for topic 20 (“internet addiction”), the following abstract from the paper by Ni et al. (2009) is one of three most representative documents:

“The prevalence of Internet addiction and influential factors associated with Internet addiction among freshmen college students were investigated in this study. A total of 3,557 first-year university students from a university in northwest China were surveyed with Young's 20-item Internet Addiction Test (IAT) questionnaire, a Self-Rating Depression scale (SDS), a Self-Rating Anxiety scale (SAS), and a basic information questionnaire. A rate of 6.44% of the participants surveyed showed Internet addiction. The students with Internet addiction had higher scores of SDS and SAS compared with those without Internet addiction (*p* < 0.01). There were significant positive correlations between SDS and SAS scores and Internet addiction (*p* < 0.001). Multiple logistic regression analyses showed that a single-parent family, the age of first exposure to Internet use, the age of the student, city residence, and homesickness were significantly associated with Internet addiction (*p* < 0.01). Special and closer attention should be paid to these factors, and a risk-focus approach should be implemented in university freshmen with depression, anxiety, and other influential factors associated with Internet addiction at the beginning of their university life to guarantee the fulfillment of their academic study and graduation.”

Supplementary Table 1 presents the list of the topics with the following information: the custom label of the topic and its classification score; the number of documents in the topic; and a representative document. The latter is provided as a citation with the DOI, so that the abstract (and the full text of the paper, if available) can be easily accessed.

**Figure 3:**
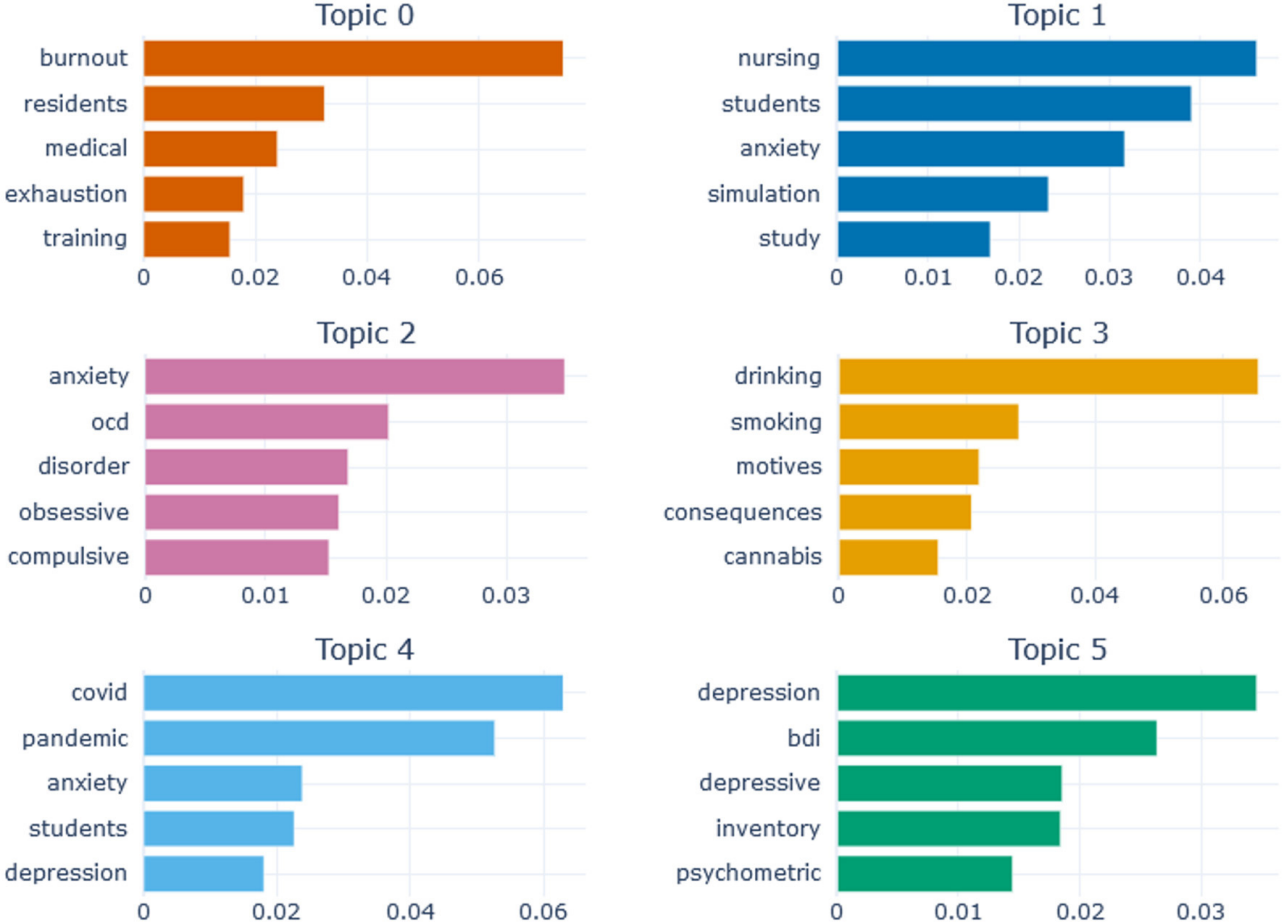
Topic representations for the first six topics. OCD is Obsessive-Compulsive Disorder, BDI is Beck Depression Inventory. The x-axis shows c-TF-IDF scores of words.

Topics were explored in regard to their word representations, as shown in Figure 3. For the sake of visual clarity, only the first six topics are presented in the plot as an example. These topics include 1,087 articles, which is 38 % of the total sample. The first five most representative terms are shown for each topic, and the horizontal axis shows their c-TF-IDF scores. Thus, the first task, describing the topics themselves, was completed, the topics were labeled, the relevant words and their c-TF-IDF scores explored, the relevant documents obtained.

**Figure 4:**
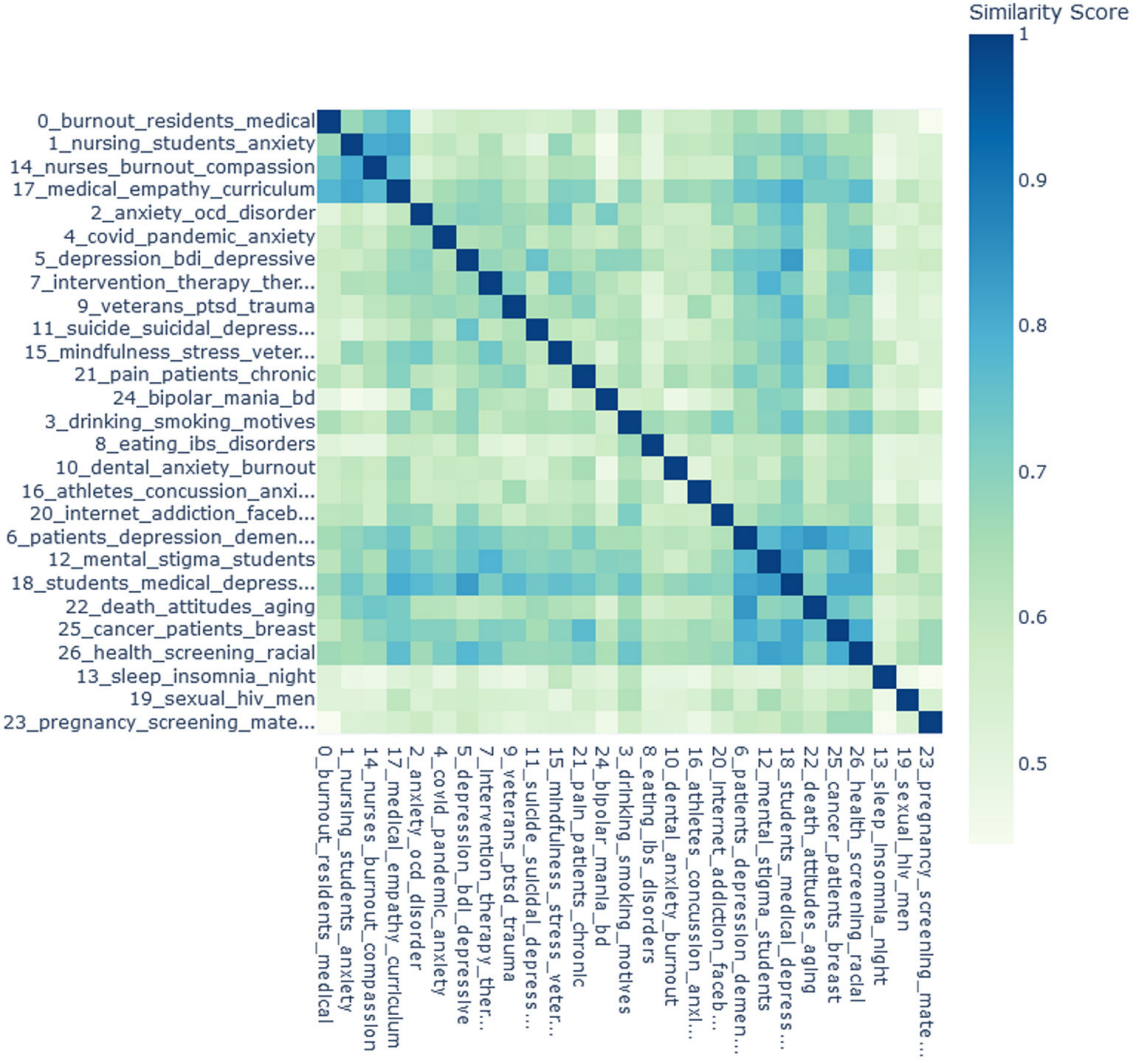
The heatmap for the 27 topics. The topics are sorted for clearer presentation. The scores are the cosine similarities between the topic embeddings. Darker colors represent higher similarity scores.

The relationships between topics were explored with the document embedding visualizations (not presented in the paper, the interested researcher is referred to the code), the heatmap, and the cluster dendrogram. In Figure 4, the heatmap is shown. The topics are sorted for clearer presentation of similarities between them. In the first group of topics, similarity scores between topic 0 (“medical residents' burnout”), topic 1 (“nursing students' anxiety and skills”), topic 14 (“burnout in nursing”), and topic 17 (“medical curriculum and empathy”) range from 0.67 to 0.82. Overall, some topics are more isolated than others. It could be seen, for instance, that topic 18 (“medical students' depression”) has relatively high similarity scores with a number of other topics, while topic 13 (“sleep problems”) has very low similarity scores with other topics.

**Figure 5:**
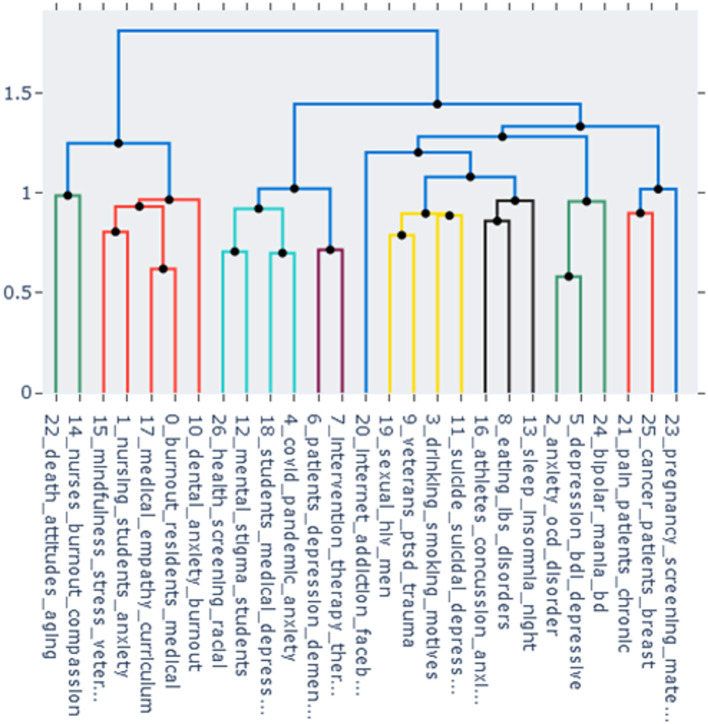
The dendrogram of the topics. The vertical axis shows the distance between clusters. Clusters are colored at the threshold of 1.

In Figure 5, agglomeration levels of the topics are presented as a cluster dendrogram. The vertical axis shows the distance between clusters, so it can be seen at what distance clusters merge. Different colors are used for different clusters starting from distance 1 (the default option).

Some topics have relatively high cosine similarity according to the heatmap and are agglomerated at the early stage of clustering in the dendrogram, such as topic 0 (“medical residents' burnout”) and topic 17 (“medical curriculum and empathy”). Other topics are close according to the dendrogram but not to the heatmap. For example, topic 5 (“psychometrics of depression”) forms a cluster with topic 2 (“OCD and anxiety”) in the dendrogram, but in the heatmap has the highest similarity score with topic 11 (“suicidality and depression”).

**Figure 6:**
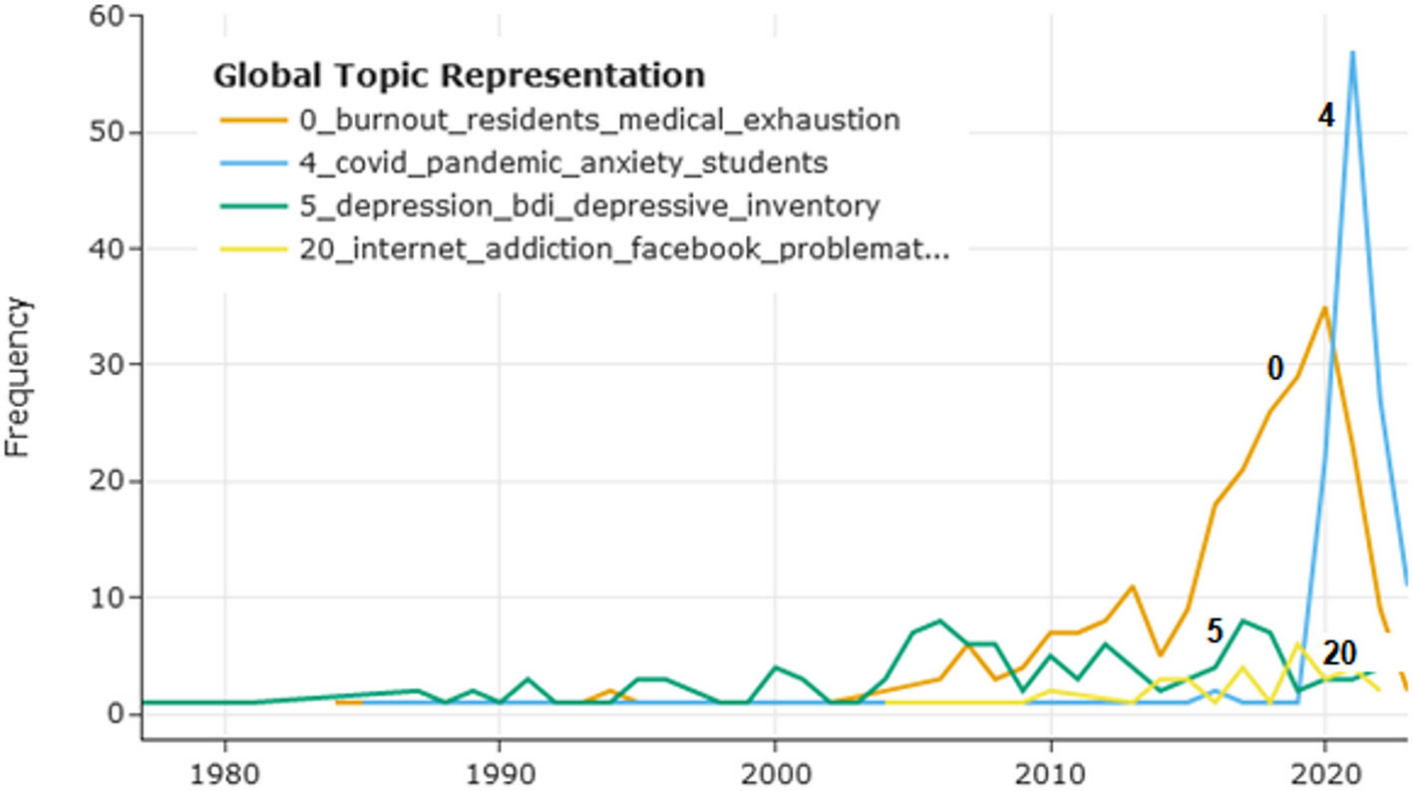
The number of publications on topics 0, 4, 5, and 20 changing with time. The x-axis shows the time and the y-axis the number of publications.

Dynamic topic modeling in the frame of BERTopic allows tracing evolution of topics with time. Figure 6 depicts the timeline for topics 0 (“medical residents' burnout”), 4 (“pandemic-related anxiety”), 5 (“psychometrics of depression”), and 20 (“internet addiction”), with the vertical axis showing the number of publications on a certain topic. The topics were selected based on their impact on the field and potential interest to researchers. Topic 4 (“pandemic-related anxiety”) predictably peaked in years 2020–2021, with topic 0 (“medical residents' burnout”) following it, while publications on topic 5 (“psychometrics of depression”) and topic 20 (“internet addiction”) were comparatively scarce. In the graph, years 2022–2023 might be underrepresented, as the papers published in these recent years are still being added to the database.

**Figure 7:**
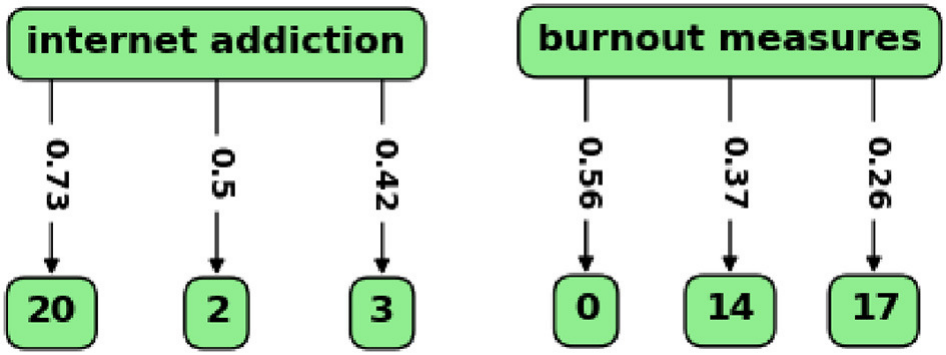
Relevant topics and similarity scores for two search terms. The topics are shown as the nodes, and similarity scores between the term and the topics are on the edges.

Finally, the BERTopic function that finds topics most similar to a search term was used, and the results were visualized with the custom graph. In Figure 7, the results for two search terms are shown, in each case with three relevant topics as nodes and similarity scores on the edges from the term to the topics.

With the term “internet addiction”, the researcher can see how topic 20 (“internet addiction”) is related to other topics. It appeared that topic 2 (“OCD and anxiety”) and topic 3 (“substance abuse”) are also relevant to this term, though with lower similarity scores. With the term “burnout measures”, the researcher can find topics related to psychometric instruments for burnout. These are topics 0 (“medical residents' burnout”), topic 14 (“burnout in nursing”), and topic 17 (“medical curriculum and empathy”). These results are discussed in the next section.

## 5 Discussion

Mental health in academia is a problem that requires attention from the research community. Depression, anxiety, and burnout are distinct but interrelated conditions highly prevalent in academic environments, and they are increasingly addressed by scientific publications. With growing number of these papers, methods of extracting meaningful insights from previous findings should be used that can process large amounts of data and employ human expertise. In this study, BERTopic, an advanced topic modeling technique, was applied to analyzing the abstracts of PubMed articles on depression, anxiety, and burnout in academia. Comparing to literature reviews that are conducted by human experts, topic modeling as a machine learning method gives a possibility to deal with a substantially larger number of sources: in this study, 2,846 scientific papers published from 1975 to 2023 were explored.

The study illustrated the use of the modular structure of BERTopic, which provides researchers with sufficient flexibility for building models that satisfy their requirements. In this study, the model was selected based on topic interpretability, the proportion of outliers, and TC and TD as supplementary criteria. The undeniable subjectivity of the topic interpretability criterion can be understood, from the methodological point of view, as the need to be aware of multiple perspectives on topic selection depending on the specific research context (Gelman and Hennig, 2017). In practical terms, however sophisticated the method of topic modeling is, the researcher's domain knowledge still plays a crucial role in model selection and interpretation (Egger and Yu, 2022; Grootendorst, 2022). The analytical decisions made in this study were transparently reported. They included data collection (selecting the query and the database); choice of specific BERTopic modules (such as clustering with HDBSCAN); tuning the parameters (such as minimal cluster size and MMR); and criteria for model evaluation. In model interpretation, as reiterated below, inevitable subjectivity of the researcher's perspective is even more prominent, be that labeling the topics, selecting the topics to be explored in more detail, etc.

The final BERTopic model identified 27 topics in the data, and document embeddings with hierarchical levels give a general picture of the field with relative sizes of topics (see the code for the interactive visualization). These results are presented in Supplementary Table 1, which reports the number of documents in each topic, the topic labels, and representative documents. Topics related to the concept of burnout were prominent, and from topic evolution in Figure 6 we can see that texts on burnout of medical professionals in the pandemic times influenced this situation to a substantial degree. Multiple topics were related to anxiety, out of which topic 2 (“OCD and anxiety”) might be interesting for clinicians, as its most relevant words include anxious (“phobia”) and comorbid (“OCD”) disorders. In this topic, as well as in many others, at least two of three conditions under scrutiny (in this case, “anxiety” and “depression”) are identified as the most relevant words. Depression is mentioned in a number of topics, including topic 11 (“suicidality and depression”), which could be important for researchers and clinicians. Overall, the topics identified by the model represent the field comprehensively. It can be seen from the labels of the topics that include various comorbid conditions, from OCD to bipolar disorder, substance abuse, eating disorders, etc. The number of texts addressing mental health of medical students and residents was disproportionally large in comparison to texts related to other populations in academia, which is possibly explained by the PubMed specialization.

In addition to the general overview of the field, in which regard topic modeling is similar to a literature review, though on a substantially larger scale, the researcher can focus on specific topics and explore their relevant documents, similarities to other topics, and evolution with time. This focus is also subjective to some degree; the researcher studying the results of the model might be interested in topics related to OCD or borderline disorder. In this study, the author's attention was drawn to psychometric measurement and internet-related problems. Topic 5 (“psychometrics of depression”) contains abstracts of papers, many of which discuss psychometric measures for depression, validation of new scales, or translation and adaptation of the most frequently used scale, BDI, into different languages. The importance of valid and reliable psychometric instruments for depression, anxiety, and burnout should not be underestimated. For instance, in a meta-analysis (Koutsimani et al., 2019), results of studies on burnout differed substantially depending on which burnout measure was used. In this model, BERTopic did not assign distinct topics for psychometric studies on anxiety and burnout, but these could have been included in other topics (as Figure 7 shows for burnout). For topic 5 (“psychometrics of depression”), evolution of the topic shown in Figure 6 does not reflect its potential importance for the field. This result could be biased by insufficient presentation of psychometric research on depression in PubMed, or by assignment of such papers to other topics, or by the fact that most recent papers have not been added to the database yet. However, it should be emphasized that psychometric instrument validation is the basis for methodologically rigorous research and thus needs to receive sufficient attention in the area of mental health. As it was shown that internet-related problems intertwined with depression and anxiety have become more prevalent in recent years (Servidio et al., 2021; Gavurova et al., 2022), the author also focused on topic 20 (“internet addiction”). Its relationships with other topics are elucidated with the dendrogram in Figure 5, where this topic clusters at a later stage with topics related to substance abuse, sleep problems, etc. In Figure 7, topic 20 is the most similar to the search term “internet addiction”, which is predictable considering the fact that this collocation is the topic label with the classification score 0.998; two other topics (2 and 3) present a certain interest as they could be also related to the internet addiction problem. Topic 20 included only 35 studies, and its timeline in Figure 6 did not show substantial progress in the recent years. The same considerations about bias as for topic 5 might apply, and there might be studies on internet-related problems in academia that were not included in the model. However, the importance and prevalence of internet-related problems in our digitalized world tend to increase, and focusing on this area might be beneficial for mental health research.

Limitations of the study are related to each stage of the process and can be addressed by further research. The stage of data collection includes analytical choices regarding the database, the query, and the texts themselves, which provide ample possibility for change. In this study, scientific papers were represented by abstracts, and further research might compare this with full text mining. The abstracts were extracted from PubMed, which is a relevant database containing the increasing number of scientific publications; however, other databases might be queried in the future to obtain a more representative picture of the field. The query used in this study might be tuned to maximize the coverage of literature and decrease the risk of gathering incomplete and noisy data. In this study, such academic groups as undergraduate students, doctoral candidates, and postdocs were not differentiated, and this approach can be changed in future research. At the stage of data analysis with BERTopic, the user encounters the following issues that can be addressed by further research. As mentioned above, BERTopic assigns each document to a single topic, although it is partly resolved by soft clustering of HDBSCAN (Grootendorst, 2022). A more serious problem, typical for other topic modeling methods such as LDA (Agrawal et al., 2018), is caused by the model stability issue. In BERTopic, the instability is related to the stochastic nature of the UMAP algorithm. Therefore, results of three runs for each model were averaged, and fixing a random seed in UMAP was required for reproducibility of the final model (Grootendorst, 2023).

To conclude, the study showed the use of BERTopic for analyzing literature on mental health in academia. Advantages of this novel method of topic modeling were illustrated, and limitations of the study were outlined. The findings described in this paper might shed light on areas to be further addressed by topic modeling and by mental health research. Cumulative effort of scientists in different fields can hopefully result in better procedures and instruments for diagnosing, preventing, and treating depression, anxiety, and burnout in academia.

## Data availability statement

Publicly available datasets were analyzed in this study. This data can be found here: PubMed database (https://pubmed.ncbi.nlm.nih.gov/).

## Author contributions

OL: Conceptualization, Data curation, Formal analysis, Methodology, Software, Visualization, Writing—original draft, Writing—review & editing.

## References

[B1] Abbe A. Grouin C. Zweigenbaum P. Falissard B. (2016). Text mining applications in psychiatry: a systematic literature review: text mining applications in Psychiatry. Int. J. Methods Psychiatr. Res. 25, 86–100. 10.1002/mpr.1481 26184780 PMC6877250

[B2] Agrawal A. Fu W. Menzies T. (2018). What is wrong with topic modeling? And how to fix it using search-based software engineering. Inform. Softw. Technol. 98, 74–88. 10.1016/j.infsof.2018.02.005

[B3] Albalawi R. Yeap T. H. Benyoucef M. (2020). Using topic modeling methods for short-text data: a comparative analysis. Front. Artif. Intellig. 3, 42. 10.3389/frai.2020.00042 33733159 PMC7861298

[B4] Alghamdi R. Alfalqi K. (2015). A survey of topic modeling in text mining. Int. J. Adv. Comp. Sci. Appl. 6, 5. 10.14569/IJACSA.2015.060121

[B5] Andrade D. Ribeiro I. J. S. Máté O. (2023). Academic burnout among master and doctoral students during the COVID-19 pandemic. Sci. Rep. 13, 4745. 10.1038/s41598-023-31852-w 36959340 PMC10034888

[B6] APA (2013). Diagnostic and Statistical Manual of Mental Disorders (5th ed.).

[B7] APA (2023). What are Anxiety Disorders? Available online at: https://www.psychiatry.org/patients-families/anxiety-disorders (accessed July, 2023).

[B8] Balhatchet B. Schütze H. Williams N. Ashford B. (2023). Factors that impact burnout and psychological wellbeing in Australian postgraduate medical trainees: a systematic review. Global Surg. Educ. 2, 65. 10.1007/s44186-023-00143-3 PMC8464131 34560887

[B9] Blair S. J. Bi Y. Mulvenna M. D. (2020). Aggregated topic models for increasing social media topic coherence. Appl. Intellig. 50, 138–156. 10.1007/s10489-019-01438-z

[B10] Blei D. Ng A. Jordan M. (2003). Latent dirichlet allocation. J. Mach. Learn. Res. 3. 601–608. 10.5555/944919.944937

[B11] Buddhitha P. Inkpen D. (2023). Multi-task learning to detect suicide ideation and mental disorders among social media users. Front. Res. Metrics Analyt. 8, 1152535. 10.3389/frma.2023.1152535 37138946 PMC10149941

[B12] Cahill B. (2023). Researcher Mental Health: From Raising Awareness to Providing Evidence of Best Practices. Brussels, Belgium. Zenodo: EURAXESS BHO Meeting 2023.

[B13] Campello R. J. G. B. Moulavi D. Sander J. (2013). “Density-based clustering based on hierarchical density estimates,” in Advances in Knowledge Discovery and Data Mining. PAKDD 2013. Lecture Notes in Computer Science, eds. J. Pei, V. S. Tseng, L. Cao, H. Motoda, G. Xu, G. Berlin, Heidelberg: Springer.

[B14] Cowan T. Rodriguez Z. B. Granrud O. E. Masucci M. D. Docherty N. M. Cohen A. S. (2022). Talking about health: a topic analysis of narratives from individuals with schizophrenia and other serious mental illnesses. Behav. Sci. 12, 286. 10.3390/bs12080286 36004857 PMC9405157

[B15] Cuijpers P. Vogelzangs N. Twisk J. Kleiboer A. Li J. Penninx B. W. (2014). Comprehensive meta-analysis of excess mortality in depression in the general community versus patients with specific illnesses. Am. J. Psychiatry 171, 453–462. 10.1176/appi.ajp.2013.13030325 24434956

[B16] Daenekindt S. Huisman J. (2020). Mapping the scattered field of research on higher education. A correlated topic model of 17,000 articles, 1991–2018. Higher Educ. 80, 571–587. 10.1007/s10734-020-00500-x

[B17] de Groot M. Aliannejadi M. Haas M. R. (2022). “Experiments on Generalizability of BERTopic on Multi-Domain Short Text (arXiv:2212.08459),” in arXiv. Available online at: http://arxiv.org/abs/2212.08459 (accessed July, 2023).

[B18] Devlin J. Chang M.-W. Lee K. Toutanova K. (2019). “BERT: pre-training of deep bidirectional transformers for language understanding,” in Proceedings of NAACL-HLT 2019 (Minneapolis, MN: Association for Computational Linguistics), 4171–4186.

[B19] Dyrbye L. N. Thomas M. R. Shanafelt T. D. (2006). Systematic review of depression, anxiety, and other indicators of psychological distress among U.S. and Canadian Medical Students: Academic. Medicine 81, 354–373. 10.1097/00001888-200604000-00009 16565188

[B20] Egger R. Yu J. (2022). A topic modeling comparison between LDA, NMF, Top2Vec, and BERTopic to demystify Twitter posts. Front. Sociol. 7, 886498. 10.3389/fsoc.2022.886498 35602001 PMC9120935

[B21] Gallagher R. J. Reing K. Kale D. Ver Steeg G. (2017). Anchored correlation explanation: topic modeling with minimal domain knowledge. Trans. Assoc. Computat. Linguist. 5, 529–542. 10.1162/tacl_a_00078

[B22] Gavurova B. Khouri S. Ivankova V. Rigelsky M. Mudarri T. (2022). Internet addiction, symptoms of anxiety, depressive symptoms, stress among higher education students during the COVID-19 pandemic. Front. Public Health 10, 893845. 10.3389/fpubh.2022.893845 35774570 PMC9237380

[B23] Gelman A. Hennig C. (2017). Beyond subjective and objective in statistics. J. Royal Statist. Soc. 180, 967–1033. 10.1111/rssa.12276

[B24] Grootendorst M. (2022). “BERTopic: Neural topic modeling with a class-based TF-IDF procedure (arXiv:2203.05794),” in arXiv. Available online at: http://arxiv.org/abs/2203.05794 (accessed July, 2023).

[B25] Grootendorst M. (2023). “BERTopic,” in GitHub. Available online at: https://maartengr.github.io/BERTopic/index.html (accessed July, 2023).

[B26] Guthrie S. Lichten C. A. van Belle J. Ball S. Knack A. Hofman J. (2017). Understanding Mental Health in the Research Environment: a Rapid Evidence Assessment. Santa Monica, CA: RAND Corporation. Available online at: https://www.rand.org/pubs/research_reports/RR2022.html (accessed July, 2023). 10.7249/RR2022 PMC5873519 29607246

[B27] Hannigan T. R. Haans R. F. J. Vakili K. Tchalian H. Glaser V. L. Wang M. S.. (2019). Topic modeling in management research: rendering new theory from textual data. Acad. Manage. Annals 13, 586–632. 10.5465/annals.2017.0099

[B28] Hirschfeld R. M. (2001). The comorbidity of major depression and anxiety disorders: recognition and management in primary care. Prim. Care Companion J. Clin. Psychiatry 3, 244–254. 10.4088/PCC.v03n0609 15014592 PMC181193

[B29] Ibrahim A. K. Kelly S. J. Adams C. E. Glazebrook C. (2013). A systematic review of studies of depression prevalence in university students. J. Psychiatr. Res. 47, 391–400. 10.1016/j.jpsychires.2012.11.015 23260171

[B30] Koutsimani P. Montgomery A. Georganta K. (2019). The relationship between burnout, depression, and anxiety: a systematic review and meta-analysis. Front. Psychol. 10, 284. 10.3389/fpsyg.2019.00284 30918490 PMC6424886

[B31] Kukushkin K. Ryabov Y. Borovkov A. (2022). Digital twins: a systematic literature review based on data analysis and topic modeling. Data 7, 173. 10.3390/data7120173

[B32] Lee H. Hong Lee S. Re Lee K. Hyun Kim J. (2023). ESG discourse analysis through BERTopic: comparing news articles and academic papers. Comp. Mat. Continua 75, 6023–6037. 10.32604/cmc.2023.039104

[B33] Maslach C. Schaufeli W. B. Leiter M. P. (2001). Job burnout. Annu. Rev. Psychol. 52, 397–422. 10.1146/annurev.psych.52.1.397 11148311

[B34] McInnes L. Healy J. Astels S. (2017). Hdbscan: Hierarchical density based clustering. J. Open Source Softw. 2, 11. 10.21105/joss.00205

[B35] McInnes L. Healy J. Saul N. Groberger L. (2018). UMAP: uniform manifold approximation and projection. J. Open Source Softw. 3, 861. 10.21105/joss.00861

[B36] Mirza A. A. Baig M. Beyari G. M. Halawani M. A. Mirza A. A. (2021). Depression and anxiety among medical students: a brief overview. Adv. Med. Educ. Pract. 12, 393–398. 10.2147/AMEP.S302897 33911913 PMC8071692

[B37] Moßburger L. Wende F. Brinkmann K. Schmidt T. (2020). “Exploring Online Depression Forums via Text Mining: A Comparison of Reddit and a Curated Online Forum,” in Proceedings of the 5th Social Media Mining for Health Applications (#SMM4H) Workshop & Shared Task (Barcelona: Association for Computational Linguistics), 70–81.

[B38] Müller A. (2020). Mental health disorders: prevalent but widely ignored in academia? J. Physiol. 598, 1279–1281. 10.1113/JP279386 32003870

[B39] Munthuli A. Pooprasert P. Klangpornkun N. Phienphanich P. Onsuwan C. Jaisin K.. (2023). Classification and analysis of text transcription from Thai depression assessment tasks among patients with depression. PLoS ONE 18, e0283095. 10.1371/journal.pone.0283095 36996118 PMC10062633

[B40] Ni X. Yan H. Chen S. Liu Z. (2009). Factors influencing internet addiction in a sample of freshmen university students in China. Cyberpsychol. Behav. 12, 327–330. 10.1089/cpb.2008.0321 19445631

[B41] Oveh R. O. Adewunmi M. A. Aziken G. O. (2022). “BERTopic modeling with P53 in ovarian cancer,” in 2022 5th Information Technology for Education and Development (ITED) (Abuja: IEEE), 1–4.

[B42] Park S. Kim-Knauss Y. Sim J. (2021). Leveraging text mining approach to identify what people want to know about mental disorders from online inquiry platforms. Front. Public Health 9, 759802. 10.3389/fpubh.2021.759802 34712643 PMC8546111

[B43] Reisenbichler M. Reutterer T. (2019). Topic modeling in marketing: Recent advances and research opportunities. J. Busin. Econ. 89, 327–356. 10.1007/s11573-018-0915-7

[B44] Röder M. Both A. Hinneburg A. (2015). “Exploring the Space of Topic Coherence Measures,” in Proceedings of the Eighth ACM International Conference on Web Search and Data Mining (New York, NY: Association for Computing Machinery), 399–408.

[B45] Ryan E. Hore K. Power J. Jackson T. (2023). The relationship between physician burnout and depression, anxiety, suicidality and substance abuse: a mixed methods systematic review. Front. Public Health 11, 1133484. 10.3389/fpubh.2023.1133484 37064688 PMC10098100

[B46] Sánchez-Franco M. J. Calvo-Mora A. Periáñez-Cristobal R. (2023). Clustering abstracts from the literature on Quality Management (1980–2020). Total Qual. Management & Busin. Excel. 34, 959–989. 10.1080/14783363.2022.2139674

[B47] Schmidt B. M. (2013). Words alone: dismantling topic models in the humanities. J. Digit. Humanit. 2, 1.

[B48] Servidio R. Bartolo M. G. Palermiti A. L. Costabile A. (2021). Fear of COVID-19, depression, anxiety, and their association with Internet addiction disorder in a sample of Italian students. J. Affect. Disord. Rep. 4, 100097. 10.1016/j.jadr.2021.100097

[B49] Shatte A. B. R. Hutchinson D. M. Teague S. J. (2019). Machine learning in mental health: a scoping review of methods and applications. Psychol. Med. 49, 1426–1448. 10.1017/S0033291719000151 30744717

[B50] Svärdman F. Sjöwall D. Lindsäter E. (2022). Internet-delivered cognitive behavioral interventions to reduce elevated stress: a systematic review and meta-analysis. Intern. Intervent. 29, 100553. 10.1016/j.invent.2022.100553 35781929 PMC9240371

[B51] Turian J. Ratinov L. Bengio Y. (2010). “Word representations: A simple and general method for semi-supervised learning,” in Proceedings of the 48th Annual Meeting of the Association for Computational Linguistics, ACL '10, 384–394.

[B52] Udupa A. Adarsh K. N. Aravinda A. Godihal N. H. Kayarvizhy N. (2022). “An exploratory analysis of GSDMM and BERTopic on short text topic modeling,” in 2022 Fourth International Conference on Cognitive Computing and Information Processing (CCIP), 1–9.

[B53] Vogt L. Konrad M. Prinz M. (2023). Towards a Rosetta Stone for (meta)Data: Learning From Natural Language to Improve Semantic and Cognitive Interoperability.

[B54] Winer E. S. Bryant J. Bartoszek G. Rojas E. Nadorff M. R. Kilgore J. (2017). Mapping the relationship between anxiety, anhedonia, and depression. J. Affect. Disord. 221, 289–296. 10.1016/j.jad.2017.06.006 28668590 PMC6080718

[B55] World Health Organization (2023). Depression [Fact Sheet]. Available online at: http://www.who.int/en/news-room/fact-sheets/detail/depression

[B56] Yang Y. Sun H. Zhang Y. Zhang T. Gong J. Wei Y.. (2021). Dimensionality reduction by UMAP reinforces sample heterogeneity analysis in bulk transcriptomic data. Cell Rep. 36, 109442. 10.1016/j.celrep.2021.109442 34320340

[B57] Zhang T. Schoene A. M. Ji S. Ananiadou S. (2022). Natural language processing applied to mental illness detection: a narrative review. NPJ Digital Med. 5, 46. 10.1038/s41746-022-00589-7 PMC8993841 35396451

