# Supplementary material for: Depression, anxiety, and burnout in academia: topic modeling of PubMed abstracts
Source: Front Res Metr Anal. 2023 Nov 27;8:1271385. doi: 10.3389/frma.2023.1271385 (PMC10711630; doi:10.3389/frma.2023.1271385)
Supplement: Supplementary file 1 [file Table_1.docx]

**Supplementary Table 1. Topics, custom labels with classification scores, number of documents in each topic, and references to representative documents.**

| **N** | **Topic label ( score)** | **Documents** | **A representative document** |
| --- | --- | --- | --- |
| 0 | Medical residents’ burnout (.996) | 238 | Dyrbye, L. N., Thomas, M. R., Huntington, J. L., Lawson, K. L., Novotny, P. J., Sloan, J. A., & Shanafelt, T. D. (2006). Personal life events and medical student burnout: a multicenter study. *Academic medicine: journal of the Association of American Medical Colleges*, *81*(4), 374–384. <https://doi.org/10.1097/00001888-200604000-00010> |
| 1 | Nursing students' anxiety and skills (.993) | 223 | Najjar, R. H., Lyman, B., & Miehl, N. (2015). Nursing students' experiences with high-fidelity simulation. *International journal of nursing education scholarship, 12, /j/ijnes.2015.12.issue-1/ijnes-2015-0010/ijnes-2015-0010.xml.* <https://doi.org/10.1515/ijnes-2015-0010> |
| 2 | OCD and anxiety (.995) | 203 | Mojsa-Kaja, J., Golonka, K., & Gawłowska, M. (2016). Preliminary analyses of psychometric characteristics of the Polish version of the Obsessive-Compulsive Inventory-Revised (OCI-R) in a non-clinical sample. *International journal of occupational medicine and environmental health, 29*(6), 1011–1021. <https://doi.org/10.13075/ijomeh.1896.00792> |
| 3 | Substance abuse (.996) | 148 | Hefner, K. R., Sollazzo, A., Mullaney, S., Coker, K. L., & Sofuoglu, M. (2019). E-cigarettes, alcohol use, and mental health: Use and perceptions of e-cigarettes among college students, by alcohol use and mental health status. *Addictive behaviors, 91*, 12–20. <https://doi.org/10.1016/j.addbeh.2018.10.040> |
| 4 | Pandemic-related anxiety (.996) | 129 | Alsolais, A., Alquwez, N., Alotaibi, K. A., Alqarni, A. S., Almalki, M., Alsolami, F., Almazan, J., & Cruz, J. P. (2021). Risk perceptions, fear, depression, anxiety, stress and coping among Saudi nursing students during the COVID-19 pandemic*. Journal of mental health (Abingdon, England), 30*(2), 194–201. <https://doi.org/10.1080> |
| 5 | Psychometrics of depression (.991) | 120 | Ignjatović-Ristić, D., Hinić, D., & Jović, J. (2012). Evaluation of the Beck Depression Inventory in a nonclinical student sample. *The West Indian medical journal, 61*(5), 489–493. <https://doi.org/10.7727/wimj.2011.215> |
| 6 | Depression in elderly patients (.993) | 105 | Tuna Doğrul, R., Doğan Varan, H., Cemal Kızılarslanoğlu, M., Kılıç, M. K., Kara, Ö., Arık, G., Halil, M. G., Cankurtaran, M., & Doğu, B. B. (2021). Association of physical frailty with cognitive function and mood in older adults without dementia and depression. *Turkish journal of medical sciences, 51*(5), 2334–2340. <https://doi.org/10.3906/sag-2011-277> |
| 7 | Anxiety therapy and interventions (.991) | 89 | Bendtsen, M., Müssener, U., Linderoth, C., & Thomas, K. (2020). A Mobile Health Intervention for Mental Health Promotion Among University Students: Randomized Controlled Trial. *JMIR mHealth and uHealth, 8*(3), e17208. <https://doi.org/10.2196/17208> |
| 8 | Eating disorders and depression (.996) | 79 | Tavolacci, M. P., Déchelotte, P., & Ladner, J. (2020). Eating Disorders among College Students in France: Characteristics, Help-and Care-Seeking. *International journal of environmental research and public health, 17*(16), 5914. <https://doi.org/10.3390/ijerph17165914> |
| 9 | Posttraumatic stress and depression (.997) | 78 | Othieno, C. J., Okoth, R., Peltzer, K., Pengpid, S., & Malla, L. O. (2015). Traumatic experiences, posttraumatic stress symptoms, depression, and health-risk behavior in relation to injury among University of Nairobi students in Kenya. *International journal of psychiatry in medicine, 50*(3), 299–316. <https://doi.org/10.1177/0091217415610310> |
| 10 | Burnout in dentistry (.994) | 74 | Blumer, S., Peretz, B., Yukler, N., & Nissan, S. (2020). Dental Anxiety, Fear and Anxiety of Performing Dental Treatments among Dental Students during Clinical Studies. *The Journal of clinical pediatric dentistry, 44*(6), 407–411. <https://doi.org/10.17796/1053-4625-44.6.3> |
| 11 | Suicidality and depression (.997) | 72 | Tyssen, R., Vaglum, P., Grønvold, N. T., & Ekeberg, O. (2001). Suicidal ideation among medical students and young physicians: a nationwide and prospective study of prevalence and predictors. *Journal of affective disorders, 64*(1), 69–79. <https://doi.org/10.1016/s0165-0327(00)00205-6> |
| 12 | Stigma of mental disorders in students (.994) | 67 | Golberstein, E., Eisenberg, D., & Gollust, S. E. (2008). Perceived stigma and mental health care seeking. *Psychiatric services (Washington, D.C.), 59*(4), 392–399. <https://doi.org/10.1176/ps.2008.59.4.392> |
| 13 | Sleep problems (.994) | 66 | Wong, M. L., Lau, E. Y., Wan, J. H., Cheung, S. F., Hui, C. H., & Mok, D. S. (2013). The interplay between sleep and mood in predicting academic functioning, physical health and psychological health: a longitudinal study. *Journal of psychosomatic research, 74*(4), 271–277. <https://doi.org/10.1016/j.jpsychores.2012.08.014> |
| 14 | Burnout in nursing (.994) | 60 | Boamah, S. A., & Laschinger, H. (2016). The influence of areas of worklife fit and work-life interference on burnout and turnover intentions among new graduate nurses. *Journal of nursing management, 24*(2), E164–E174. <https://doi.org/10.1111/jonm.12318> |
| 15 | Mindfulness interventions for depression and anxiety (.989) | 58 | O'Driscoll, M., Sahm, L. J., Byrne, H., Lambert, S., & Byrne, S. (2019). Impact of a mindfulness-based intervention on undergraduate pharmacy students' stress and distress: Quantitative results of a mixed-methods study. *Currents in pharmacy teaching & learning, 11*(9), 876–887. <https://doi.org/10.1016/j.cptl.2019.05.014> |
| 16 | Anxiety and depression in sports (.992) | 56 | Yang, J., Peek-Asa, C., Corlette, J. D., Cheng, G., Foster, D. T., & Albright, J. (2007). Prevalence of and risk factors associated with symptoms of depression in competitive collegiate student athletes. *Clinical journal of sport medicine: official journal of the Canadian Academy of Sport Medicine, 17*(6), 481–487. <https://doi.org/10.1097/JSM.0b013e31815aed6b> |
| 17 | Medical curriculum and empathy (.997) | 55 | Assing Hvidt, E., Søndergaard, J., Hvidt, N. C., Wehberg, S., Büssing, A., & Andersen, C. M. (2020). Development in Danish medical students' empathy: study protocol of a cross-sectional and longitudinal mixed-methods study. *BMC medical education, 20*(1), 54. <https://doi.org/10.1186/s12909-020-1967-2> |
| 18 | Medical students’ depression (.991) | 51 | Koly, K. N., Sultana, S., Iqbal, A., Dunn, J. A., Ryan, G., & Chowdhury, A. B. (2021). Prevalence of depression and its correlates among public university students in Bangladesh. *Journal of affective disorders, 282*, 689–694. <https://doi.org/10.1016/j.jad.2020.12.137> |
| 19 | Sexuality and depression (.991) | 45 | Wang, Y. C., Miao, N. F., & Chang, S. R. (2021). Internalized homophobia, self-esteem, social support and depressive symptoms among sexual and gender minority women in Taiwan: An online survey. *Journal of psychiatric and mental health nursing, 28*(4), 601–610. <https://doi.org/10.1111/jpm.12705> |
| 20 | Internet addiction (.998) | 35 | Ni, X., Yan, H., Chen, S., & Liu, Z. (2009). Factors influencing internet addiction in a sample of freshmen university students in China*. Cyberpsychology & behavior: the impact of the Internet, multimedia and virtual reality on behavior and society, 12*(3), 327–330. <https://doi.org/10.1089/cpb.2008.0321> |
| 21 | Chronic pain and depression (.997) | 35 | Lövgren, M., Gustavsson, P., Melin, B., & Rudman, A. (2014). Neck/shoulder and back pain in new graduate nurses: A growth mixture modeling analysis. *International journal of nursing studies, 51*(4), 625–639. <https://doi.org/10.1016/j.ijnurstu.2013.08.009> |
| 22 | Death attitudes anxiety (.991) | 33 | Thorson, J. A., & Powell, F. C. (1991). Medical students' attitudes towards ageing and death: a cross-sequential study. *Medical education, 25*(1), 32–37. <https://doi.org/10.1111/j.1365-2923.1991.tb00023.x> |
| 23 | Postnatal depression and anxiety (.996) | 33 | Fay, K. E., & Yee, L. M. (2020). Birth Outcomes Among Women Affected by Reproductive Coercion. *Journal of midwifery & women's health, 65*(5), 627–633. <https://doi.org/10.1111/jmwh.13107> |
| 24 | Bipolar and schizophrenic disorders (.998) | 32 | Fisk, C., Dodd, A. L., & Collins, A. (2015). Response styles, bipolar risk, and mood in students: The Behaviours Checklist. *Psychology and psychotherapy, 88*(4), 412–426. <https://doi.org/10.1111/papt.12052> |
| 25 | Coping with cancer (.994) | 31 | Karibayeva, I., Turdaliyeva, B., Zainal, N. Z., Bagiyarova, F., & Kussainova, D. (2022). Prevalence of Anxiety Symptoms in Women Newly Diagnosed with Breast Cancer in Kazakhstan and Its Associated Factors. *Asian Pacific journal of cancer prevention: APJCP, 23*(8), 2813–2819. <https://doi.org/10.31557/APJCP.2022.23.8.2813> |
| 26 | Discrimination in health care (.993) | 31 | Hardeman, R. R., Perry, S. P., Phelan, S. M., Przedworski, J. M., Burgess, D. J., & van Ryn, M. (2016). Racial Identity and Mental Well-Being: The Experience of African American Medical Students, A Report from the Medical Student CHANGE Study. *Journal of racial and ethnic health disparities, 3*(2), 250–258. <https://doi.org/10.1007/s40615-015-0136-5> |
